# 4-(Dimethyl­amino)pyridinium tri­bromido­{3-[bromo/hydro­(0.9/0.1)]-4-(dimethyl­amino)pyridine-κ*N* ^1^}cobaltate(II)

**DOI:** 10.1107/S1600536809027913

**Published:** 2009-07-22

**Authors:** Kong Mun Lo, Seik Weng Ng

**Affiliations:** a Department of Chemistry, University of Malaya, 50603 Kuala Lumpur, Malaysia

## Abstract

The reaction of a cobalt(II) salt with 4-(dimethyl­amino)pyridinium hydro­bromide perbromide yielded the title compound, (C_7_H_11_N_2_)[CoBr_3_(C_7_H_9.1_Br_0.9_N_2_)]. In the anion, the Co^II^ atom is coordinated in a distorted tetra­hedral geometry by three Br atoms and the pyridine N atom of a bromine-substituted 4-(dimethyl­amino)pyridine mol­ecule, whose formation probably results from an incomplete substitution (90%) catalysed by the Co^II^ ion. One of the three bromine atoms bonded to the metal is disordered over two sites in a 0.9:0.1 ratio. An N—H⋯Br hydrogen bond connects the cation and anion.

## Related literature

For bis­(4-(dimethyl­amino)pyridinium) tetra­bromidocobaltate, see: Lo & Ng (2009 ▶). For other trihalocobaltate(II) anions having a pyridine-type donor ligand, see: Bogdanović *et al.* (2001 ▶); Crane *et al.* (2004 ▶); Divjaković *et al.* (1982 ▶); Hahn *et al.* (1997 ▶); Mueller-Westerhoff *et al.* (1996 ▶); Sumner & Steinmetz (1985 ▶).
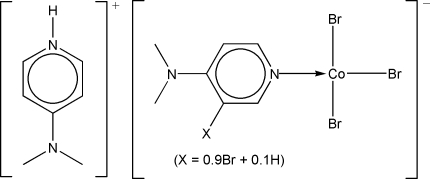


## Experimental

### 

#### Crystal data

(C_7_H_11_N_2_)[CoBr_3_(C_7_H_9.1_Br_0.9_N_2_)]
*M* *_r_* = 615.02
Triclinic, 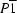

*a* = 8.3768 (2) Å
*b* = 10.2622 (2) Å
*c* = 12.4691 (3) Å
α = 99.028 (2)°
β = 98.927 (1)°
γ = 106.933 (2)°
*V* = 989.57 (4) Å^3^
*Z* = 2
Mo *K*α radiation
μ = 8.74 mm^−1^
*T* = 150 K
0.40 × 0.20 × 0.10 mm

#### Data collection

Bruker APEXII CCD diffractometer
Absorption correction: multi-scan (*SADABS*; Sheldrick, 1996 ▶) *T*_min_ = 0.128, *T*_max_ = 0.475 (expected range = 0.112–0.417)
7974 measured reflections
4451 independent reflections
3019 reflections with *I* > 2σ(*I*)
*R*_int_ = 0.053

#### Refinement

*R*[*F* ^2^ > 2σ(*F* ^2^)] = 0.046
*wR*(*F* ^2^) = 0.130
*S* = 0.97
4451 reflections
221 parameters
7 restraints
H-atom parameters constrained
Δρ_max_ = 1.29 e Å^−3^
Δρ_min_ = −1.04 e Å^−3^

### 

Data collection: *APEX2* (Bruker, 2007 ▶); cell refinement: *SAINT* (Bruker, 2007 ▶); data reduction: *SAINT*; program(s) used to solve structure: *SHELXS97* (Sheldrick, 2008 ▶); program(s) used to refine structure: *SHELXL97* (Sheldrick, 2008 ▶); molecular graphics: *X-SEED* (Barbour, 2001 ▶); software used to prepare material for publication: *publCIF* (Westrip, 2009 ▶).

## Supplementary Material

Crystal structure: contains datablocks global, I. DOI: 10.1107/S1600536809027913/hy2210sup1.cif

Structure factors: contains datablocks I. DOI: 10.1107/S1600536809027913/hy2210Isup2.hkl

Additional supplementary materials:  crystallographic information; 3D view; checkCIF report

## Figures and Tables

**Table 1:** Selected bond lengths (Å)

Co1—Br1	2.4086 (11)
Co1—Br2	2.3958 (10)
Co1—Br3	2.3814 (13)
Co1—Br3′	2.376 (9)
Co1—N1	2.032 (5)

**Table 2:** Hydrogen-bond geometry (Å, °)

*D*—H⋯*A*	*D*—H	H⋯*A*	*D*⋯*A*	*D*—H⋯*A*
N3—H3⋯Br1	0.88	2.74	3.434 (6)	137

**Fig. 1.**
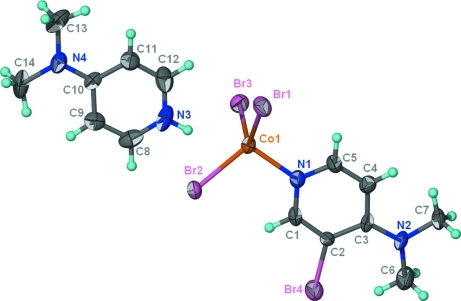
Molecular structure of the title compound. Displacement ellipsoids are drawn at the 70% probability level. Minor disordered sites are omitted for clarity.

## supplementary crystallographic information

### Comment

The reaction of cobalt(II) nitrate and 4-(dimethylamino)pyridinium hydrobromide perbromide yields bis(4-(dimethylamino)pyridinium) tetrabromidocobaltate (Lo & Ng, 2009). A similar reaction with cobalt acetate in place of cobalt nitrate yields a new 4-(dimethylamino)pyridinium salt, [C_7_H_11_N_2_][CoBr_3_(C_7_H_9.1_Br_0.9_N_2_)] (Scheme 1, Fig. 1). The Co^II^ atom is coordinated by a bromine-substituted 4-(dimethylamino)pyridine molecule, whose formation probably results from an incomplete (90%) electrophilic substitution of 4-(dimethylamino)pyridine that is probabably catalyzed by the cobaltous ion.

### Experimental

Green cobalt acetate (0.70 g, 2.8 mmol) dissolved in water (2 ml) and 4-(dimethylamino)pyridinium hydrobromide perbromide (1.00 g, 2.8 mmol) dissolved in ethanol (50 ml) were mixed and the mixture was heated for one hour. The red solution was filtered; well-formed deep-blue crystals were isolated from the solution after several days.

### Refinement

H atoms were placed at calculated positions (C—H = 0.95 and 0.98, N—H = 0.88 Å) and were treated as riding on their parent atoms, with *U*_iso_(H) = 1.2(or 1.5)*U*_eq_(C).

One of the three Br atoms that are bonded to Co1 is disordered over two positions (Br3 and Br3'). The Br3 atom is 3.5 Å from Br4^i^ atom [symmetry code: (i) = 1-x, 1-y, 2-z]. However, as the Br3' atom is only 3.0 Å from Br4^i^, the atom that is linked to the C2 atom should then be a mixture of Br and H atoms, with the provision that the occupancies of the Br3 and Br4 atoms are identical. As the occupancies refined to nearly 0.9:0.1, the occupancy factors were then fixed as 0.90 and 0.1 for Br3 and Br3', as well as for Br4 and H2. Other ratios, e.g. 0.85:0.15 and 0.95:0.05, gave less satisfactory *R* indices and large peaks/deep holes in the difference Fourier map. The anisotropic displacement of the minor occupant was restrained to be nearly isotropic; the Co–Br distances were restrained to within 0.01 Å of each other.

The final difference Fourier map had a peak in the vicinity of Br2 and a hole in the vicinty of Br4. The magnitudes of both could be decreased by lowering the 2θ limit to 50°.

### Crystal data

**Table:**

(C_7_H_11_N_2_)[CoBr_3_(C_7_H_9.1_Br_0.9_N_2_)]	*Z* = 2
*M**_r_* = 615.02	*F*(000) = 591.2
Triclinic, *P*1	*D*_x_ = 2.064 Mg m^−^^3^
Hall symbol: -P 1	Mo *K*α radiation, λ = 0.71073 Å
*a* = 8.3768 (2) Å	Cell parameters from 6987 reflections
*b* = 10.2622 (2) Å	θ = 2.4–28.3°
*c* = 12.4691 (3) Å	µ = 8.74 mm^−^^1^
α = 99.028 (2)°	*T* = 150 K
β = 98.927 (1)°	Block, brown
γ = 106.933 (2)°	0.40 × 0.20 × 0.10 mm
*V* = 989.57 (4) Å^3^	

### Data collection

**Table:**

Bruker APEXII CCD diffractometer	4451 independent reflections
Radiation source: fine-focus sealed tube	3019 reflections with *I* > 2σ(*I*)
graphite	*R*_int_ = 0.053
φ and ω scans	θ_max_ = 27.5°, θ_min_ = 1.7°
Absorption correction: multi-scan (*SADABS*; Sheldrick, 1996)	*h* = −10→10
*T*_min_ = 0.128, *T*_max_ = 0.475	*k* = −12→13
7974 measured reflections	*l* = −16→16

### Refinement

**Table:**

Refinement on *F*^2^	Primary atom site location: structure-invariant direct methods
Least-squares matrix: full	Secondary atom site location: difference Fourier map
*R*[*F*^2^ > 2σ(*F*^2^)] = 0.046	Hydrogen site location: inferred from neighbouring sites
*wR*(*F*^2^) = 0.130	H-atom parameters constrained
*S* = 0.97	*w* = 1/[σ^2^(*F*_o_^2^) + (0.0634*P*)^2^] where *P* = (*F*_o_^2^ + 2*F*_c_^2^)/3
4451 reflections	(Δ/σ)_max_ = 0.001
221 parameters	Δρ_max_ = 1.29 e Å^−^^3^
7 restraints	Δρ_min_ = −1.04 e Å^−^^3^

### Fractional atomic coordinates and isotropic or equivalent isotropic displacement parameters (Å^2^)

**Table:**

	*x*	*y*	*z*	*U*_iso_*/*U*_eq_	Occ. (<1)
Br1	0.26488 (9)	0.68358 (8)	0.54136 (5)	0.0375 (2)	
Br2	0.64945 (8)	0.59464 (7)	0.69989 (5)	0.03080 (18)	
Br3	0.49021 (18)	0.89737 (12)	0.84679 (11)	0.0315 (3)	0.90
Br3'	0.531 (2)	0.8775 (13)	0.8695 (11)	0.068 (5)	0.10
Br4	0.30233 (9)	0.25573 (8)	0.96716 (6)	0.0350 (2)	0.90
Co1	0.41492 (10)	0.67837 (9)	0.72097 (7)	0.0271 (2)	
N1	0.2478 (6)	0.5344 (5)	0.7796 (4)	0.0280 (12)	
N2	−0.0965 (6)	0.2792 (5)	0.9321 (4)	0.0276 (12)	
N3	0.6361 (7)	0.7246 (6)	0.4462 (5)	0.0414 (15)	
H3	0.5580	0.6723	0.4751	0.050*	
N4	1.0006 (7)	0.9742 (6)	0.3157 (4)	0.0318 (12)	
C1	0.2977 (8)	0.4510 (6)	0.8403 (5)	0.0267 (13)	
H1	0.4135	0.4540	0.8492	0.032*	
C2	0.1936 (7)	0.3618 (6)	0.8904 (5)	0.0226 (12)	
H2'	0.2368	0.3026	0.9293	0.027*	0.10
C3	0.0191 (7)	0.3572 (6)	0.8846 (5)	0.0248 (13)	
C4	−0.0282 (8)	0.4425 (7)	0.8152 (5)	0.0320 (15)	
H4	−0.1438	0.4404	0.8015	0.038*	
C5	0.0825 (8)	0.5267 (7)	0.7677 (6)	0.0338 (15)	
H5	0.0420	0.5830	0.7239	0.041*	
C6	−0.0620 (10)	0.1971 (10)	1.0125 (8)	0.063 (3)	
H6A	0.0449	0.2503	1.0660	0.094*	
H6B	−0.0516	0.1101	0.9737	0.094*	
H6C	−0.1559	0.1756	1.0518	0.094*	
C7	−0.2699 (8)	0.2868 (7)	0.9146 (6)	0.0377 (16)	
H7A	−0.3213	0.2596	0.8349	0.057*	
H7B	−0.2661	0.3824	0.9433	0.057*	
H7C	−0.3385	0.2234	0.9537	0.057*	
C8	0.7904 (10)	0.7112 (7)	0.4577 (5)	0.0362 (16)	
H8	0.8148	0.6441	0.4962	0.043*	
C9	0.9138 (9)	0.7919 (7)	0.4155 (5)	0.0320 (15)	
H9	1.0236	0.7811	0.4254	0.038*	
C10	0.8813 (7)	0.8921 (6)	0.3568 (4)	0.0240 (13)	
C11	0.7136 (9)	0.9012 (7)	0.3454 (5)	0.0360 (16)	
H11	0.6826	0.9655	0.3062	0.043*	
C12	0.5987 (9)	0.8172 (8)	0.3908 (6)	0.0405 (17)	
H12	0.4871	0.8243	0.3831	0.049*	
C13	0.9613 (11)	1.0765 (8)	0.2567 (6)	0.051 (2)	
H13A	0.9162	1.1366	0.3038	0.076*	
H13B	0.8758	1.0282	0.1881	0.076*	
H13C	1.0654	1.1334	0.2384	0.076*	
C14	1.1733 (9)	0.9648 (8)	0.3293 (6)	0.048 (2)	
H14A	1.2214	0.9768	0.4084	0.073*	
H14B	1.2452	1.0380	0.2994	0.073*	
H14C	1.1689	0.8733	0.2893	0.073*	

### Atomic displacement parameters (Å^2^)

**Table:**

	*U*^11^	*U*^22^	*U*^33^	*U*^12^	*U*^13^	*U*^23^
Br1	0.0327 (4)	0.0541 (5)	0.0345 (4)	0.0205 (3)	0.0109 (3)	0.0188 (3)
Br2	0.0220 (3)	0.0369 (4)	0.0365 (4)	0.0102 (3)	0.0092 (3)	0.0119 (3)
Br3	0.0321 (5)	0.0292 (5)	0.0328 (5)	0.0089 (4)	0.0070 (3)	0.0071 (4)
Br3'	0.076 (9)	0.060 (7)	0.057 (7)	0.001 (5)	0.034 (6)	0.001 (5)
Br4	0.0253 (4)	0.0469 (5)	0.0403 (4)	0.0144 (3)	0.0087 (3)	0.0237 (4)
Co1	0.0236 (5)	0.0288 (5)	0.0310 (5)	0.0068 (4)	0.0110 (3)	0.0108 (4)
N1	0.025 (3)	0.030 (3)	0.031 (3)	0.008 (2)	0.009 (2)	0.011 (2)
N2	0.022 (3)	0.031 (3)	0.029 (3)	0.003 (2)	0.010 (2)	0.013 (2)
N3	0.033 (3)	0.047 (4)	0.034 (3)	−0.005 (3)	0.011 (3)	0.007 (3)
N4	0.026 (3)	0.038 (3)	0.026 (3)	0.002 (2)	0.003 (2)	0.010 (2)
C1	0.017 (3)	0.033 (4)	0.028 (3)	0.007 (3)	0.007 (2)	0.002 (3)
C2	0.023 (3)	0.023 (3)	0.025 (3)	0.009 (2)	0.008 (2)	0.010 (2)
C3	0.020 (3)	0.025 (3)	0.024 (3)	0.000 (2)	0.006 (2)	0.000 (2)
C4	0.023 (3)	0.035 (4)	0.039 (4)	0.007 (3)	0.004 (3)	0.015 (3)
C5	0.024 (3)	0.039 (4)	0.046 (4)	0.014 (3)	0.011 (3)	0.020 (3)
C6	0.036 (5)	0.085 (7)	0.083 (6)	0.017 (5)	0.021 (4)	0.058 (5)
C7	0.024 (4)	0.042 (4)	0.049 (4)	0.008 (3)	0.017 (3)	0.010 (3)
C8	0.055 (5)	0.029 (4)	0.023 (3)	0.011 (3)	0.007 (3)	0.007 (3)
C9	0.036 (4)	0.031 (4)	0.027 (3)	0.012 (3)	0.001 (3)	0.002 (3)
C10	0.027 (3)	0.025 (3)	0.015 (3)	0.003 (3)	0.002 (2)	0.003 (2)
C11	0.033 (4)	0.045 (4)	0.030 (4)	0.014 (3)	0.002 (3)	0.013 (3)
C12	0.023 (4)	0.059 (5)	0.033 (4)	0.007 (3)	0.002 (3)	0.008 (3)
C13	0.060 (5)	0.035 (4)	0.050 (5)	−0.001 (4)	0.008 (4)	0.022 (4)
C14	0.031 (4)	0.056 (5)	0.045 (5)	−0.003 (4)	0.013 (3)	−0.002 (4)

### Geometric parameters (Å, °)

**Table:**

Co1—Br1	2.4086 (11)	C4—H4	0.9500
Co1—Br2	2.3958 (10)	C5—H5	0.9500
Co1—Br3	2.3814 (13)	C6—H6A	0.9800
Co1—Br3'	2.376 (9)	C6—H6B	0.9800
Br4—C2	1.888 (6)	C6—H6C	0.9800
Co1—N1	2.032 (5)	C7—H7A	0.9800
N1—C1	1.340 (8)	C7—H7B	0.9800
N1—C5	1.347 (8)	C7—H7C	0.9800
N2—C3	1.343 (7)	C8—C9	1.356 (9)
N2—C6	1.454 (9)	C8—H8	0.9500
N2—C7	1.461 (8)	C9—C10	1.416 (8)
N3—C8	1.328 (9)	C9—H9	0.9500
N3—C12	1.338 (9)	C10—C11	1.421 (9)
N3—H3	0.8800	C11—C12	1.353 (9)
N4—C10	1.333 (7)	C11—H11	0.9500
N4—C13	1.456 (9)	C12—H12	0.9500
N4—C14	1.463 (9)	C13—H13A	0.9800
C1—C2	1.368 (8)	C13—H13B	0.9800
C1—H1	0.9500	C13—H13C	0.9800
C2—C3	1.439 (8)	C14—H14A	0.9800
C2—H2'	0.9500	C14—H14B	0.9800
C3—C4	1.415 (9)	C14—H14C	0.9800
C4—C5	1.350 (9)		
			
N1—Co1—Br3'	105.5 (5)	N2—C6—H6A	109.5
N1—Co1—Br3	107.98 (15)	N2—C6—H6B	109.5
Br3'—Co1—Br3	12.5 (4)	H6A—C6—H6B	109.5
N1—Co1—Br2	106.97 (14)	N2—C6—H6C	109.5
Br3'—Co1—Br2	104.7 (4)	H6A—C6—H6C	109.5
Br3—Co1—Br2	114.67 (5)	H6B—C6—H6C	109.5
N1—Co1—Br1	105.94 (15)	N2—C7—H7A	109.5
Br3'—Co1—Br1	123.1 (4)	N2—C7—H7B	109.5
Br3—Co1—Br1	111.17 (5)	H7A—C7—H7B	109.5
Br2—Co1—Br1	109.64 (4)	N2—C7—H7C	109.5
C1—N1—C5	116.2 (5)	H7A—C7—H7C	109.5
C1—N1—Co1	122.3 (4)	H7B—C7—H7C	109.5
C5—N1—Co1	121.2 (4)	N3—C8—C9	121.1 (6)
C3—N2—C6	125.9 (5)	N3—C8—H8	119.5
C3—N2—C7	119.4 (5)	C9—C8—H8	119.5
C6—N2—C7	114.3 (5)	C8—C9—C10	120.9 (6)
C8—N3—C12	120.3 (6)	C8—C9—H9	119.5
C8—N3—H3	119.9	C10—C9—H9	119.5
C12—N3—H3	119.9	N4—C10—C9	122.3 (6)
C10—N4—C13	120.2 (6)	N4—C10—C11	121.8 (6)
C10—N4—C14	121.2 (6)	C9—C10—C11	115.9 (6)
C13—N4—C14	118.6 (6)	C12—C11—C10	119.2 (6)
N1—C1—C2	124.6 (5)	C12—C11—H11	120.4
N1—C1—H1	117.7	C10—C11—H11	120.4
C2—C1—H1	117.7	N3—C12—C11	122.6 (6)
C1—C2—C3	120.5 (5)	N3—C12—H12	118.7
C1—C2—Br4	114.0 (4)	C11—C12—H12	118.7
C3—C2—Br4	125.5 (4)	N4—C13—H13A	109.5
C1—C2—H2'	119.8	N4—C13—H13B	109.5
C3—C2—H2'	119.8	H13A—C13—H13B	109.5
N2—C3—C4	120.3 (5)	N4—C13—H13C	109.5
N2—C3—C2	127.4 (6)	H13A—C13—H13C	109.5
C4—C3—C2	112.2 (5)	H13B—C13—H13C	109.5
C5—C4—C3	123.4 (6)	N4—C14—H14A	109.5
C5—C4—H4	118.3	N4—C14—H14B	109.5
C3—C4—H4	118.3	H14A—C14—H14B	109.5
N1—C5—C4	122.9 (6)	N4—C14—H14C	109.5
N1—C5—H5	118.6	H14A—C14—H14C	109.5
C4—C5—H5	118.6	H14B—C14—H14C	109.5
			
Br3'—Co1—N1—C1	−83.8 (6)	Br4—C2—C3—C4	−176.4 (5)
Br3—Co1—N1—C1	−96.6 (5)	N2—C3—C4—C5	177.9 (6)
Br2—Co1—N1—C1	27.3 (5)	C2—C3—C4—C5	−5.0 (9)
Br1—Co1—N1—C1	144.2 (4)	C1—N1—C5—C4	1.3 (9)
Br3'—Co1—N1—C5	90.1 (7)	Co1—N1—C5—C4	−173.0 (5)
Br3—Co1—N1—C5	77.3 (5)	C3—C4—C5—N1	1.8 (11)
Br2—Co1—N1—C5	−158.8 (5)	C12—N3—C8—C9	1.0 (10)
Br1—Co1—N1—C5	−41.9 (5)	N3—C8—C9—C10	−0.6 (10)
C5—N1—C1—C2	−0.6 (9)	C13—N4—C10—C9	−179.5 (6)
Co1—N1—C1—C2	173.6 (5)	C14—N4—C10—C9	0.0 (9)
N1—C1—C2—C3	−3.0 (9)	C13—N4—C10—C11	−0.2 (9)
N1—C1—C2—Br4	178.6 (5)	C14—N4—C10—C11	179.3 (6)
C6—N2—C3—C4	−174.2 (7)	C8—C9—C10—N4	179.1 (6)
C7—N2—C3—C4	−2.0 (9)	C8—C9—C10—C11	−0.3 (9)
C6—N2—C3—C2	9.1 (10)	N4—C10—C11—C12	−178.6 (6)
C7—N2—C3—C2	−178.7 (6)	C9—C10—C11—C12	0.7 (9)
C1—C2—C3—N2	−177.7 (6)	C8—N3—C12—C11	−0.5 (10)
Br4—C2—C3—N2	0.5 (9)	C10—C11—C12—N3	−0.3 (11)
C1—C2—C3—C4	5.4 (8)		

### Hydrogen-bond geometry (Å, °)

**Table:**

*D*—H···*A*	*D*—H	H···*A*	*D*···*A*	*D*—H···*A*
N3—H3···Br1	0.88	2.74	3.434 (6)	137
